# To Explore the Key Subgroup and Their Immune Microenvironment During the Formation of Coronary Plaque With scRNA-seq

**DOI:** 10.1155/crp/3221767

**Published:** 2025-05-24

**Authors:** Xinhan Li, Qiulai Li, Haiying Liu, Ying Zhang, Jie Xia, Xin Wang, Tao Lei, Jun Ma

**Affiliations:** ^1^Shandong University of Traditional Chinese Medicine, Jinan 250014, China; ^2^Yantai Hospital of Traditional Chinese Medicine, Yantai 264001, China; ^3^No. 970 Hospital of the PLA Joint Logistics Support Force, Yantai 264001, China

**Keywords:** aging pathway, coronary plaque, NK cells, single-cell RNA sequencing, transcription factors

## Abstract

**Background:** The most important pathological basis of coronary heart disease is atheroma formation. If atheromatous plaque occurs and is not treated promptly and effectively, the plaque will gradually grow, causing the lumen of the coronary arteries to gradually narrow until it is completely occluded, causing angina pectoris and even myocardial infarction, but its cellular heterogeneity is not fully understood.

**Methods:** We utilized various techniques including single-cell RNA sequencing, CytoTRACE, monocle, slingshot, CellChat, and SCENIC to investigate the significant subgroup of NK cells in 15 specimens from individuals in order to understand their contributions to the development of coronary plaque.

**Results:** The analysis revealed that studying the subgroup C1 RACK1+ NK cells was crucial for this paper. We investigated its effect on coronary plaque and then analyzed C1 RACK1+ NK cells to explore the expression of this subgroup in pseudotime trajectories, cell interactions, and transcription factors.

**Conclusion:** Single-cell RNA sequencing could provide a deeper understanding of the factors that have an important impact on the development of coronary plaque, improved the understanding of the microenvironment of coronary plaque, provided enlightenment for the treatment of coronary plaque in the future, and helped to improve the diagnosis of coronary plaque and design the best treatment strategy.

## 1. Introduction

Cardiovascular diseases (CVD), such as coronary artery disease (CAD), are the leading cause of death and morbidity worldwide, with 1 death per 5 patients [1, 2]. Atherosclerosis is the main characteristic of CAD, a chronic inflammatory condition of the blood vessels caused by the buildup of cholesterol-rich lipoproteins, resulting in vascular damage, inflammation, and plaque development. This chronic plaque accumulation in artery walls can result in thrombosis, heart attacks, or strokes [3–5]. The formation of atherosclerosis is a complex and long-term process involving multiple cellular and molecular mechanisms [6], Cited Pages. In the initial stage, vascular endothelial cells are stimulated by risk factors and their function changes, becoming prone to allow lipid components in the blood to enter the underlining of blood vessels. Monocytes are then recruited into the subintima and differentiate into macrophages, which ingests large amounts of oxidized low-density lipoprotein cholesterol through receptors on their surface and gradually transform into foam cells, an early signature event in atherosclerotic plaque formation [7]. With the progression of the disease, the plaque keeps increasing and tends to be stable [8]. A variety of cells are involved in the process of coronary plaque formation, and in-depth understanding of the heterogeneity of these cells can more accurately reveal the changes at various cellular levels in the pathogenesis of coronary heart disease, and clarify the specific roles of different cell subgroups in different stages of atherosclerosis, so as to avoid ignoring key pathogenesis links due to overall average analysis.

A major obstacle in treating patients with CAD is the presence of atherosclerotic plaques, which can lead to acute coronary syndromes by rupturing and blocking the arteries [9]. While noninvasive imaging techniques like computed tomography and magnetic resonance imaging show promise in improving our understanding of the disease, there remains a need for better access to coronary plaque at the cellular level [10–12]. Recent clinical trials have shown that anti-inflammatory medications can potentially be used in the future as treatment options for atherosclerotic plaque in addition to statin therapy [13], reducing secondary cardiovascular events [14]. Although there have been achievements, there is still a need to address the impact of systemic cytokine neutralization on plaque characteristics as well as concerns regarding compromised host defenses and infection [15, 16]. Targeting specific inflammatory pathways in affected blood vessels through topical administration is a better strategy for addressing plaque advancement than using systemic treatment, as it has the potential to enhance both effectiveness and safety. Nevertheless, the intricate nature of plaques poses a significant challenge in creating targeted approaches for treating lesions.

Natural killer (NK) cells are key components of the innate immune system and play complex roles in coronary plaque formation. From the perspective of inflammatory response, inflammation is crucial in the occurrence and development of coronary atherosclerotic lesions, and sustained inflammatory response can lead to plaque instability and increase the risk of cardiovascular events. NK cells can participate in the inflammatory cascade by secreting cytokines. In the early stage of atherosclerosis, NK cells may play a certain inhibitory role in plaque formation by killing cells infected with pathogens, reducing inflammatory stimuli. However, in some cases, such as in the presence of chronic infections with atherogenic pathogens, NK cells appear to support a pro-inflammatory environment that accelerates plaque formation and development [17].

The emergence of single-cell sequencing technology enables the examination of gene expression and regulation in disease and development at the individual cell level. For example, single-cell RNA sequencing (scRNA-seq) studies have addressed the cellular diversity and genetic characterization of atherosclerotic lesions in humans and mice [18–21]. Monocle2 algorithm utilizes the single-cell transcriptome expression matrix to imitate the biological functions of cell populations by unsupervised learning various branches of cell developmental trajectories [22]. Moreover, the uniform manifold approximation and projection (UMAP) algorithm is capable of grouping cells to identify distinct genes in various conditions and examining the key genes that impact diverse pathways of specialization. Slingshot is a unique and flexible tool that combines the highly stable technology required for complex single-cell data with the ability to identify multiple trajectories. Accurate lineage inference is a critical step in identifying dynamic temporal gene expression [23]. CellChat is a tool for single-cell data analysis to infer the intercellular communication network. Its core principle is based on ligand–receptor interactions. By integrating single-cell transcriptome data, it can identify the possible signal transduction pathways between cells. A transcription factor (TF) is a type of protein that has the ability to attach to particular DNA sequences. These proteins can either work independently or in conjunction with other proteins to enhance or inhibit the process of specific genes being brought to RNA polymerase, ultimately controlling gene expression.

The traditional bulk RNA sequencing method deals with the mixture of all cells and averages the potential differences of cell type-specific transcriptome [24]. On the other hand, scRNA-seq has the capability to examine the genetic activity of an individual cell and unravel the communication network between cells, enabling the detection of a cell's condition in various illnesses. So far, several studies using scRNA-seq have analyzed the plaque tissues of CAD patients to determine the expression of individual cells in CAD plaque-related cells [1, 25, 26]. However, the mechanism and key targets of cell–cell interaction between related cells in coronary plaque are still unclear. Therefore, the study of cell–cell interactions and immune microenvironment in coronary plaques is urgently needed to be understood, and it is also the focus of our research.

To comprehensively investigate the process of cell–cell communication and identify potential therapeutic targets for coronary plaque, scRNA-seq was utilized to analyze human coronary arteries from 12 donors and blood samples from 3 donors. The studies focused on identifying cell types involved in coronary plaque formation, with a particular emphasis on a subgroup that was found to be crucial for exploring novel treatment options. In a word, our study identified the gene expression characteristics and molecular pathways related to disease progression in each patient, which provided new insights for the future research and clinical treatment of coronary plaque.

## 2. Methods

### 2.1. Data Source

Data sourced from the GEO website (https//:www.ncbi.nlm.nih.gov/geo/) [27, 28] identified by the GSE number GSE196943.

### 2.2. Processing of scRNA-seq Data

The initial gene expression data were analyzed using Seurat software package (Version 4.3.0) [1, 29–31]. Cells of superior quality were acquired by adhering to specific standards: eliminating cells with unusually high nFeature and nCount values, ensuring that mitochondrial gene expression in a cell was under 20% of the total and that erythrocyte gene expression in a cell was under 5% of the total.

The DoubletFinder package was used to remove the doublets [32, 33]. The samples were standardized to identify the top 2000 highly variable genes and normalize their data [34]. Afterward, we conducted additional analysis on the data using PCA and applied the harmony technique to eliminate the batch effect across samples [35, 36]. And, 30 key principal components (PCs) were chosen for UMAP dimensionality reduction and gene expression visualization. Cell clusters were labeled based on the CellMarker database sourced from earlier publications [37, 38]. Afterward, we also noted the ratio of various cell categories.

### 2.3. Differentially Expressed Genes (DEGs)

DEGs for each cell type were determined using the FindAllMarker function in Seurat software by analyzing the standardized expression data, selecting genes expressed in over 25% of cells in the cluster with a logFC value above 0.25 [39].

### 2.4. Gene Ontology (GO), Kyoto Encyclopedia of Genes and Genomes (KEGG) and Gene Set Enrichment Analysis (GSEA)

Genes with adjusted *p* value < 0.05 were considered to be statistically significant in KEGG [40–42] and GO enrichment analysis [43–47]. The ClusterProfiler software package (Version 0.1.1) was utilized for the enrichment and analysis of genes that are specific to clusters [48].

GSEA is a computational technique used to assess if a predefined group of genes displays significant, consistent variations between two biological conditions [49–51].

### 2.5. Differentiation of NK Cells Based on inferCNV

The inferCNV package (https://github.com/broadinstitute/inferCNV/wiki) was utilized to differentiate NK cells by estimating the initial copy number variation (CNV) signal for each region [15]. T-cells were used as a reference group, and the subgroup with high CNV was designated as NK cells that we want to study.

### 2.6. Determination of Cell Subgroups

All NK cells were extracted and normalized again, and the top 2000 hypervariable genes were found, and their data were standardized. Afterward, we conducted additional analysis using PCA and applied the harmony technique to eliminate the batch effect among the samples. Known typical marker genes of each cell type were annotated based on cell subgroups. Cell populations exhibiting comparable gene expression profiles were classified as identical cell types and visualized on a two-dimensional map utilizing the UMAP technique.

### 2.7. Trajectory Analysis

The cell stemness of each cell subgroup was assessed using the CytoTRACE algorithm initially. CytoTRACE is capable of predicting the differentiation potential of cells and assessing where each cell is on the differentiation trajectory by calculating the cells' stemness score [52]. Clarifying the differentiation potential of cells is helpful to understand the dynamic changes of cells during coronary plaque formation. Next, we utilized the Monocle software toolkit to map out the path of cell specialization, employed DDRTree to lower the dimensionality, and examined the evolution of cell subgroups along the updated trajectory [53]. We ultimately employed the slingshot technique to delve deeper into the cell trajectory as NK cells differentiated, utilizing it to deduce cell lineage and gauge the expression level of each lineage as time progressed. Slingshot is a pseudotime series analysis tool, which is helpful to understand the progressive mechanism of plaque formation by constructing the trajectory between cells, infer the differentiation path and development process of cells, and show the state changes of cells at different stages and the dynamic changes of gene expression.

### 2.8. Intercellular Interaction Analysis

Exploring the ligand–receptor pair [54] between NK cell subgroups and cells using the CellChat package (Version 1.6.1) was necessary to analyze the cell–cell interaction network involving NK cell subgroups and other microenvironment cells [55]. We deduced intercellular communication through signal pathways and receptor–ligand interactions, investigating the coordination of signal pathways across different cell types.

### 2.9. SCENIC Analysis

SCENIC is a software designed to analyze scRNA-seq data and reconstruct gene regulatory networks to determine cell states that are consistent over time [56–58]. The study utilized the pySCENIC software package (Version 0.10.0) in Python (Version 3.7) to assess the enrichment of TFs and regulator activity. Based on co-expression and DNA motif analysis, a gene regulatory network was developed. The cellular condition was determined by analyzing the network's behavior within each individual cell. Gene motifs were ranked within a 10-kb radius of the transcription initiation site to help identify the search area for the TF regulatory network surrounding the transcription initiation site. Human gene motifs were ranked and gathered from the website https//:resources.aertslab.org/cistarget/. The Hallmark gene set of the molecular signatures database (MsigDB) was used as the database [59].

## 3. Results

### 3.1. The Primary Cellular Components Involved in the Development of Coronary Plaque

Blood samples were chosen from a public repository, human coronary arteries from 12 donors and matched blood samples from 3 donors were collected, and the primary cell types in coronary plaque were identified using scRNA-seq.

**Figure 1:**
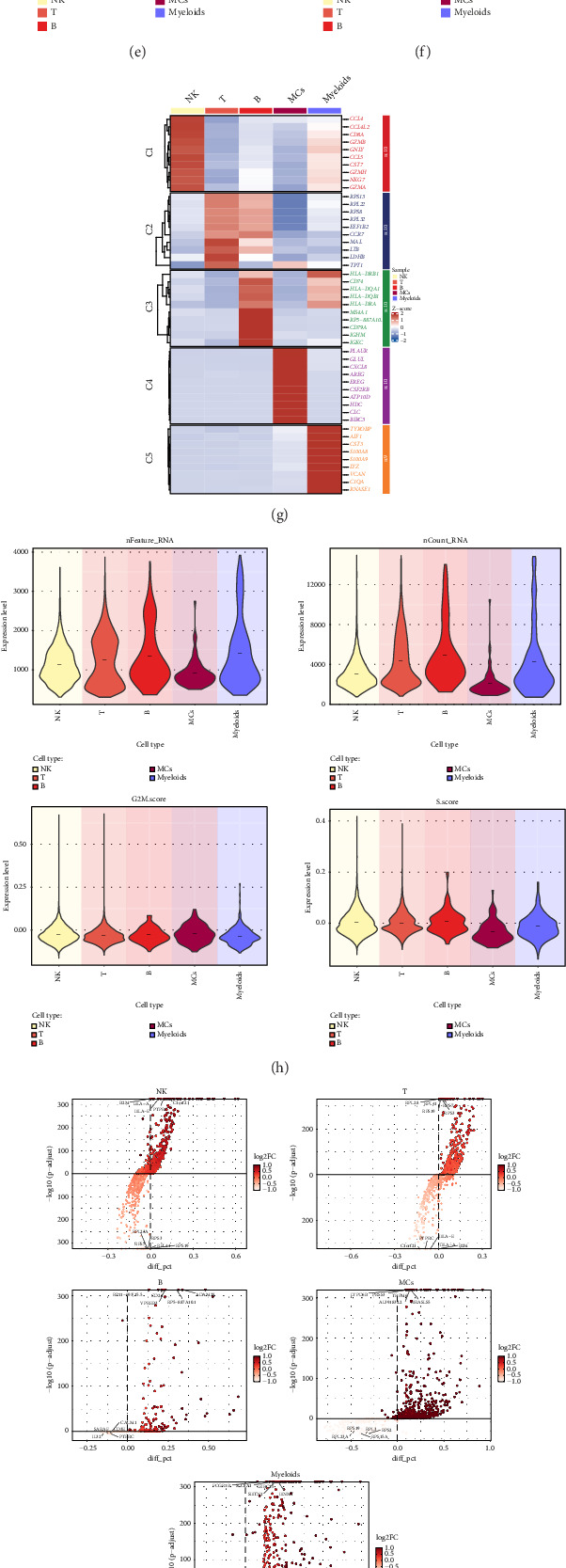
ScRNA-seq revealed the main cell types during the development of coronary plaque. (a) 3D UMAP diagram showed the distributions of five cell types in patients with coronary plaque. Each point corresponded to a single cell colored according to the cell types. (b) 3D UMAP diagram showed the distributions of two sample sources of cells from coronary plaque patients. Each point corresponded to a single cell colored according to the source of the samples. (c) 3D UMAP diagram showed the distributions of three cell cycle phases in patients with coronary plaque. Each point corresponded to a single cell colored according to phases. (d) The dot graph showed the differential expression of Top5maker genes in five different cell types of coronary plaque. The size of the dot indicated the percentage of gene expression in the cell types, and the depth of color indicated the level of gene expression. (e) The bar chart showed the proportion of five different cell types in different phases. Different colors represented different cell types. (f) The bar chart showed the proportion of five different cell types in different groups. Different colors represented different cell types. (g) Thermogram showed the expression of Top10 maker genes in five different cell types of coronary plaque. (h) Violin diagrams visualized the characteristics of five different cell types in coronary plaque: nFeature_RNA, nCount_RNA, G2M.score, and S.score. (i) Volcano plots showed the expression of differential genes in five different cell types of coronary plaque. (j) The dot diagram showed the biological processes related to five different cell types after enrichment analysis by GO-BP.

Following quality control and elimination of batch effects, a grand total of 24,420 cells were preserved. These 24,420 cells were divided into five cell types and marked with different colors. NK cells, T-cells, B-cells, MCs, and myeloids were the five cell types identified in the study (Figure 1(a)), showing the distribution of 24,420 cells across donor source groups and cell cycle phases (Figures 1(b) and 1(c)).

The highly expressed genes (top5) in each cell type were displayed using a dot graph (Figure 1(d)). Bar graphs were utilized to display the percentage of five different cell types in various cell cycle stages and donor source categories (Figures 1(e) and 1(f)). In both donor source groups and cell cycle phases, NK cells and T-cells were identified as the predominant cell types, with the proportion of NK cells in the coronary artery group being notably greater than in the peripheral blood group. Thermogram depicted the expression of top10 genes in five distinct cell varieties of coronary plaque (Figure 1(g)). At the same time, violin diagrams were used to visualize the characteristics of five different cell types in coronary plaque: nFeature_RNA, nCount_RNA, G2M.score, and S.score (Figure 1(h)). Volcano plots were utilized to display the differential gene expression in five distinct cell types of coronary plaque (Figure 1(i)). Finally, the dot plot showed the biological processes related to five different cell types after enrichment analysis by GO-BP (Figure 1(j)). NK cells, making up a significant portion, were primarily associated with biological functions like immune system process and immune process among the groups.

### 3.2. Identification of NK Cell Subgroups

**Figure 2:**
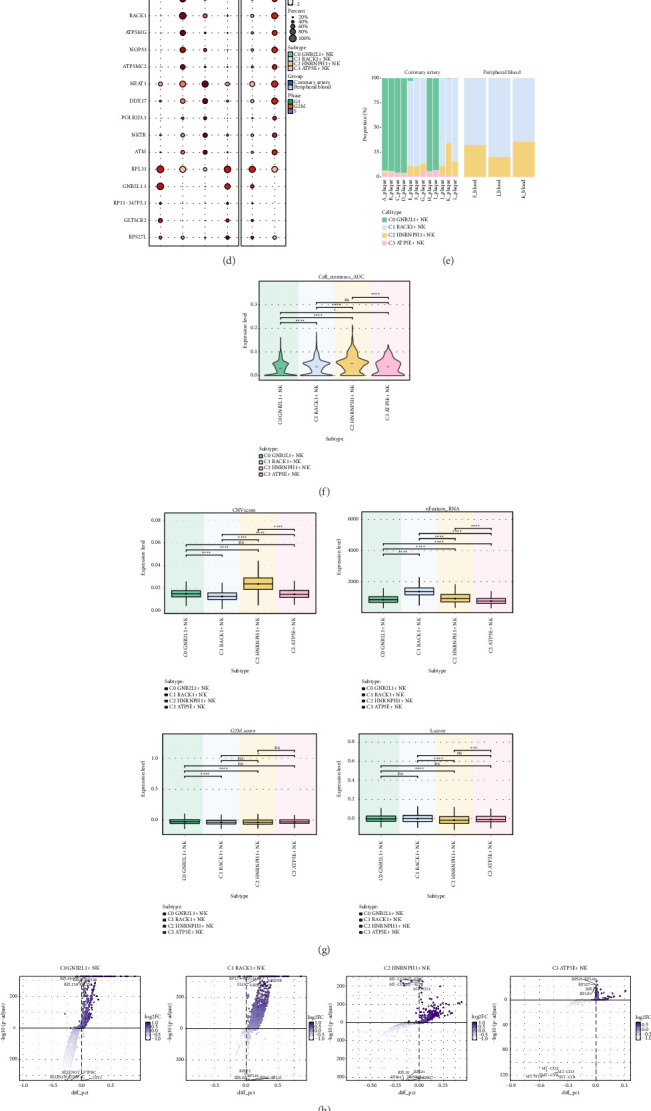
Visualization of coronary plaque cell subgroups. (a) 3D UMAP diagram showed four cell subgroups of NK cells in coronary plaque patients. (b) 3D UMAP diagram showed the distributions of two sample sources of NK cells in coronary plaque patients. Each point corresponded to a single NK cell colored according to the source of the samples. (c) 3D UMAP diagram showed the distributions of three cell cycle phases of NK cells in coronary plaque patients. Each point corresponded to a single NK cell colored according to phases. (d) Dot map showed the differential expression of Top5 maker genes in four cell subgroups. The size of the dot indicated the percentage of gene expression in the subgroups, and the depth of color indicated the level of gene expression. (e) The histogram showed the proportion of four cell subgroups in 15 donor sources. (f) Violin diagram showed the cell stemness of four cell subgroups. (g) Box diagrams visualized the relevant characteristics of four cell subgroups: CNVscore, nFeature_RNA, G2m.score, and nCount_RNA. (h) Volcano maps showed the expression of differential genes in four cell subgroups. (i) Go-BP enrichment analysis showed biological processes related to four cell subgroups.

To delve deeper into the attributes of NK cells, we employed inferCNV (Supporting Figure 1) to analyze the scRNA-seq data obtained from NK cells. A total of 11,185 NK cells were found and clustered into 4 cell subgroups: C0 *GNB2L1*+ NK cells, C1 *RACK1*+ NK cells, C2 *HNRNPH1*+ NK cells, C3 *ATP5E*+ NK cells (Figure 2(a)), and the distribution of the four cell subgroups in donor source groups and cell cycle phases are displayed (Figures 2(b) and 2(c)). The dot graph was used to describe the genes (top5) that were highly expressed in four cell subgroups of NK cells (Figure 2(d)). The bar plot was used to show the distribution of four cell subgroups from different donor sources (Figure 2(e)), and it was found that C0 *GNB2L1*+ NK cells and C3 *ATP5E*+ NK cells only existed in coronary artery group, and this suggested that the presence of NK cell subgroups in different donors was heterogeneous. The violin plot illustrating the stemness of four different cell subgroups (Figure 2(f)) revealed that the C2 *HNRNPH1*+ NK cell subgroup exhibited the greatest stemness. Several related features of CNVscore, nFeature_RNA, G2M.score, and S.score of four cell subgroups were visualized (Figure 2(g)).  Figure 2(h) volcano plots were utilized to illustrate the distinctively expressed genes among the four cell subgroups. Thermogram displayed the findings from enrichment analysis of GO-BP and differential expression of genes in four cell subgroups (Figure 2(i)). The biological processes related to C0 *GNB2L1*+ NK cells included cytoplasmic translation and ATP synthesis coupled electron transport; the biological processes related to C1 *RACK1*+ NK cells included RNA splicing and mRNA processing; and the biological processes related to C2 *HNRNPH1*+ NK cells included histone modification and RNA splicing; and the biological processes related to C3 *ATP5E*+ NK cells included cytoplasmic translation and ribosome biogenesis.

### 3.3. Visualizing the Analysis of NK Cells Over Pseudotime

**Figure 3:**
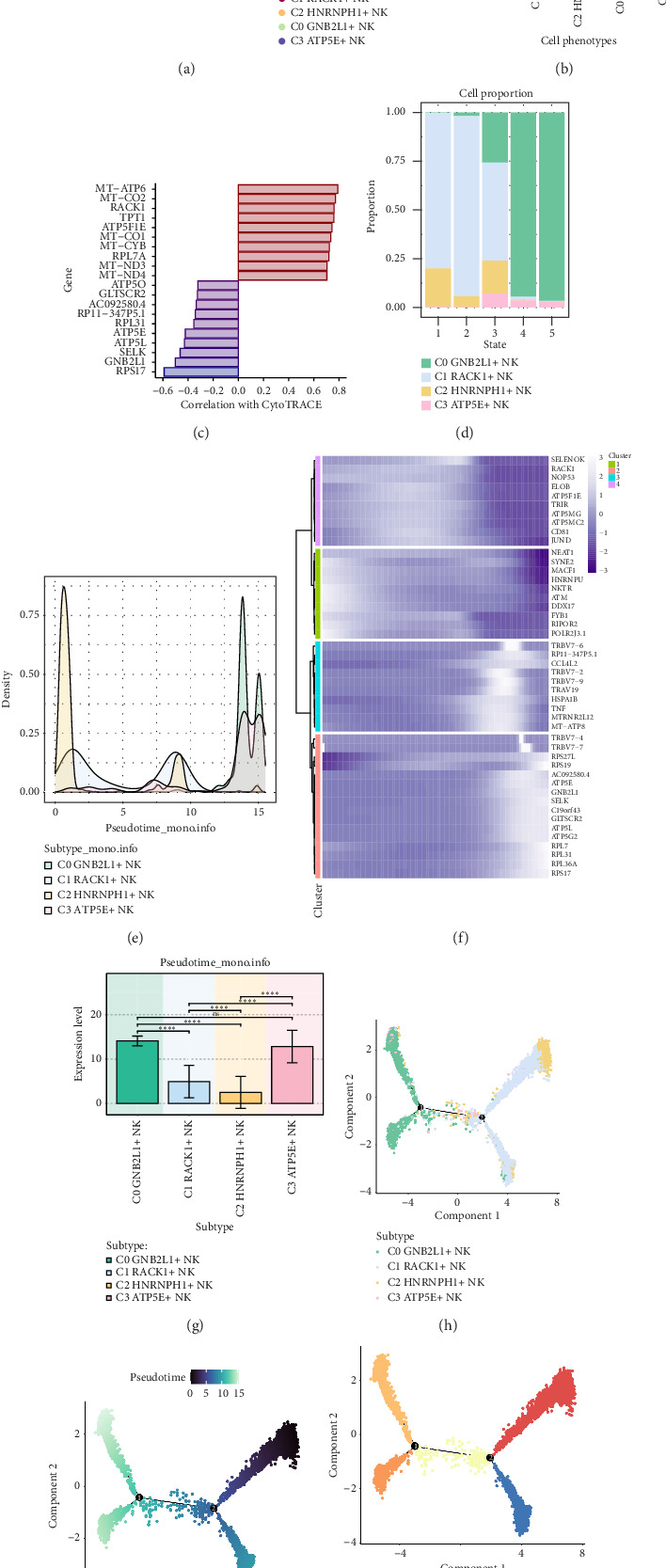
Visualization of pseudotime series analysis of NK cells by CytoTRACE and monocle. (a) The figure on the left showed the analysis of NK cell differentiation by CytoTRACE, and it was shown in the two-dimensional diagram. Colors can represent the degree of differentiation. The picture on the right showed the results of CytoTRACE displayed according to different NK cell subgroups. Colors represented different NK cell subgroups. (b) The box chart showed the predicted ordering by CytoTRACE of NK cell subgroups. (c) The bar chart showed the genes related to the cells with the highest degree of differentiation and the lowest degree of differentiation according to the correlation with CytoTRACE. (d) The histogram showed the proportion of four cell subgroups on states. (e) The ridge map showed the pseudotime series distribution of NK cell subgroups. (f) The thermogram showed the changes of marker gene in four cell subgroups with pseudotime series. (g) The histogram showed the distribution of four cell subgroups in pseudotime series. ^∗^*p* ≤ 0.05, ^∗∗^*p* < 0.01, and ^∗∗∗^*p* < 0.001 indicated significant difference, and ns indicated no significant difference. (h–j) UMAP diagram showed the distribution of four cell subgroups in pseudotime series.

The differentiation and development relationship among the four cell subgroups of NK cells were explored by analyzing the differentiation of NK cells using CytoTRACE and visualizing the results in Figure 3(a). The highest cell stemness on CytoTRACE was C1 *RACK1*+ NK cells (Figure 3(b)). Using a bar graph, the genes related to the cells with the highest degree of differentiation and the cells with the lowest degree of differentiation were displayed according to the correlation with CytoTRACE (Figure 3(c)). Among them, *RACK1* has a high correlation with CytoTRACE. The bar plot was used to show the relationship between the four cell subgroups and states (Figure 3(d)). C1 *RACK1*+ NK cells had the highest percentage in state1, state2, and state3, with C0 *GNB2L1*+ NK cells having the highest percentage in state4 and state5. And the ridge graph was used to show the expression of the four cell subgroups in pseudotime series (Figure 3(e)).

Thermogram analysis revealed that the expression changes of highly expressed genes of four cell subgroups on pseudotime series, indicating that the genes associated with the C1 *RACK1*+ NK cell subgroup were located at the beginning of the pseudotime series (Figure 3(f)).

The relationship between four cell subgroups and pseudotime series was demonstrated by box plot (Figure 3(g)). UMAP diagrams were used to show the distribution of four cell subgroups in pseudotime series. Cell subgroups differentiated from the right two branches to the left, merged at the second branch point, and continued to differentiate to the left, and then divided into two branches at the first branch point. The majority of cells in subgroups C1 and C2 were found at the start of the pseudotime series, while most cells in subgroup C0 were situated at the end point of the pseudotime series, with subgroup C3 being dispersed throughout various points on the pseudotime series (Figures 3(h), 3(i), and 3(j)). Previously noted, C1 RACK1+ NK cells had the largest percentage in state1, state2, and state3, and this group was positioned at the start of the pseudotime sequence, as evidenced by the UMAP visualization of the states.

UMAP diagrams, violin diagrams, and pseudotime series scatter diagrams were used to show the distribution of named genes of four cell subgroups in pseudotime series (Supporting Figure 2).

### 3.4. Pseudotime Trajectory of NK cell Subgroups

**Figure 4:**
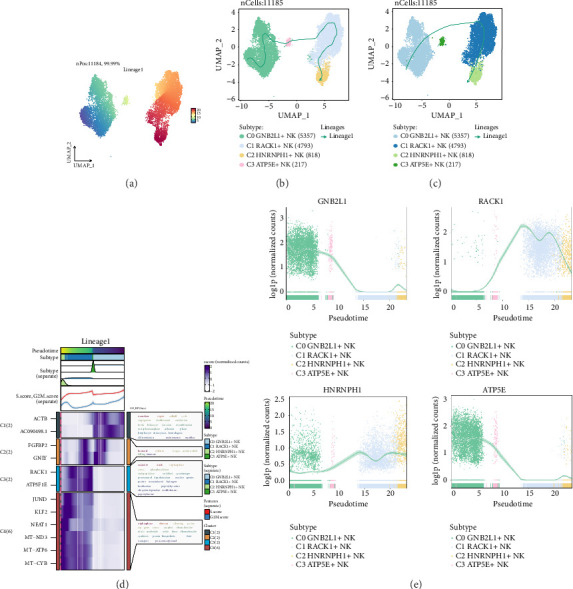
Slingshot analysis of pseudotime trajectory of NK cell subgroups. (a) UMAP diagram showed the distribution of the differentiation trajectory of NK cells by pseudotime sequence fitting. (b) UMAP diagram showed the change of lineage1 with pseudotime series. (c) UMAP diagram showed the differentiation trajectory of lineage1 in pseudotime series. (d) GO-BP enrichment analysis showed the biological processes corresponding to the pseudotime sequence trajectory of NK cell subgroups. (e) Scatter charts showed the change trajectories of the named genes of four cell subgroups of NK cells on lineage1 after visualization by slingshot.

In order to further explore whether there was a continuous branching pedigree structure in NK cells, the pseudotime trajectory of four cell subgroups were analyzed by using slingshot, and a lineage: lineage 1 was obtained. The distribution and trend of lineage1 were displayed by using UMAP diagram, and the differentiation end of lineage1 was C2 subgroup (Figures 4(a), 4(b), and 4(c)). GO-BP enrichment analysis was employed to visualize the pseudotime series trajectory of lineage1, revealing that C1 in lineage1 was associated with renal biological processes, C3 was linked to small biological processes, and C4 was connected to differentiation biological processes (Figure 4(d)).

Scatter plots were used to display the distribution of various subgroups of identified genes on lineage1 and the differentiation curve across pseudotime series (Figure 4(e)). The C1 subgroup was the only one that exhibited a notable rise in expression by the conclusion of the pseudotime sequence. Both C0 and C3 subgroups were expressed higher at the beginning of the pseudotime series and then decreased with the differentiation of the pseudotime series.

### 3.5. CellChat Analysis Between Cells

**Figure 5:**
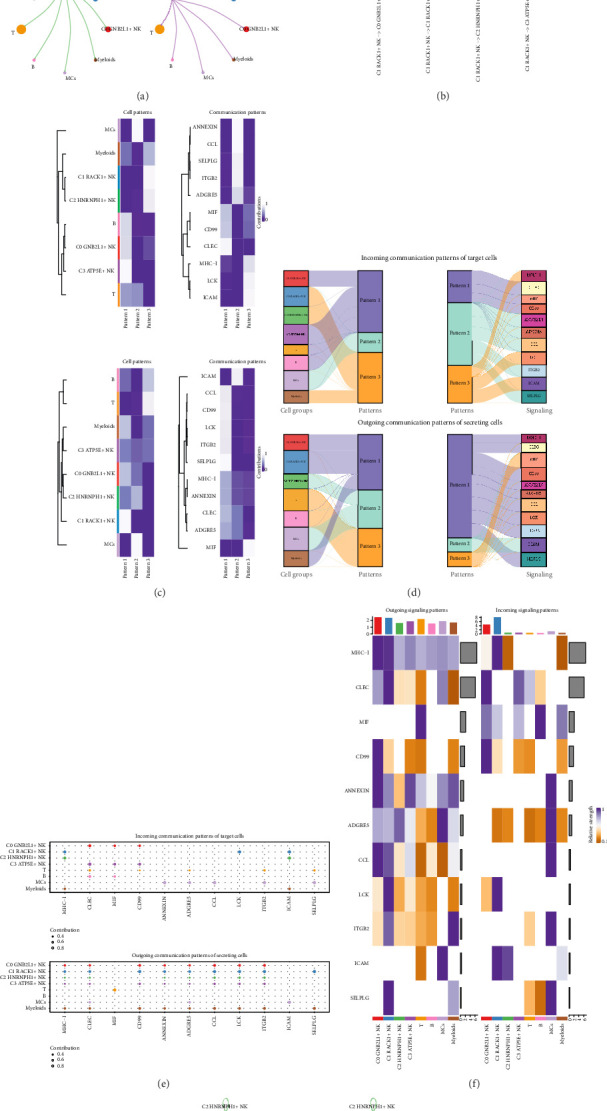
CellChat analysis among all cells. (a) Chord diagrams showed the interactions among four cell subgroups of NK cells and other cell types. (b) The dot diagram showed the ligands and receptors that interacted with C1 RACK1+ NK cells when C1 RACK1+ NK cells were set as the source. (c) The pattern recognition of incoming cells and outgoing cells in all the cells was displayed by thermogram. (d) Sankey diagrams showed the inferred outgoing communication patterns of secretory cells and showed the corresponding relationship between the inferred potential patterns and cell groups, as well as signal pathways. Top: incoming Sankey diagram; bottom: outgoing Sankey diagram. (e) Outgoing contribution bubble diagram and incoming contribution bubble diagram showed the cell communication modes between NK cells and other cells. (f) Thermogram showed the incoming and outgoing signal intensity of all cell interactions. (g) The circle diagrams showed the number (left) and intensity (right) of all cell interactions.

In order to systematically clarify complex cellular reactions and understand the interaction between cells, we explored the relationship between cells and the ligand–receptor communication network. First, we established an intercellular communication network among most cells, including four subgroups of NK cells, B-cells, T-cells, and other cells, to show the relationship between the four subgroups of NK cells and other cell types (Figure 5(a)). Several subgroups of NK cells were set as the source, and other cell types were used as targets to demonstrate their interactions. The ligand–receptor relationship of interaction between C1 *RACK1*+ NK cells and four cell subgroups was displayed by using the dot diagram when C1 *RACK1*+ NK cell subgroup was set as the source (Figure 5(b)).

We utilized CellChat's gene expression pattern analysis tools to investigate the functionality of cells and signaling pathways. Initially, we established the connection between the deduced potential communication patterns and secretory cell clusters in order to interpret the communication patterns. Three incoming signal patterns were found: pattern 1(C1 *RACK1*+ NK cells, C2 *HNRNPH1*+ NK cells, MCs), pattern 2 (C0 *GNB2L1*+ NK cells, C1 *RACK1*+ NK cells, C3 *ATP5E*+ NK cells, myeloids), and pattern 3(C3 *ATP5E*+ NK cells, T-cells, MCs), and three outgoing signal patterns: pattern 1(T-cells, MCs), pattern 2(B-cells, myeloids, C1 *RACK1*+ NK cells), and pattern 3(C0 *GNB2L1*+ NK cells, C1 *RACK1*+ NK cells, C2 *HNRNPH1*+ NK cells, MCs), each of which corresponded to some incoming and outgoing signals, respectively (Figure 5(c)).

CellChat was utilized to quantitatively assess the ligand–receptor network in four NK cell subgroups, in order to identify the crucial incoming and outgoing signals. A pattern recognition method was employed for signal prediction. NK cells had the ability for each cell type to function as either a secretory cell, sending signals, or as a target cell, receiving signals. Interactions between ligands and receptors on various cell types were expected to play a role in the formation of coronary plaque (Figure 5(e)).

Besides investigating the intricate communication within a specific pathway, a crucial concern was the coordination of functions among various cell groups and signaling pathways. CellChat utilized a non-negative matrix decomposition pattern recognition approach to uncover global communication patterns and key signals within various cell clusters for problem resolution. The application of this analysis revealed three outgoing signal patterns, three incoming signal patterns, and signal paths corresponding to different signal pathways (Figure 5(d)).

We found that LCK and MHC-I were closely related to C1 RACK1+ NK cells in both incoming and outgoing patterns (Figure 5(f)). So the next step was to discuss the relationship between the C1 *RACK1*+ NK cell subgroup and these two pathways. The calculation of interactions between the two cell types was based on the number of connections (represented by the “line” thickness) and the intensity of interactions (represented by the “line” weight). This information can be seen in Figure 5(g).

### 3.6. Visual Analysis of LCK and MHC-I Signaling Pathways

**Figure 6:**
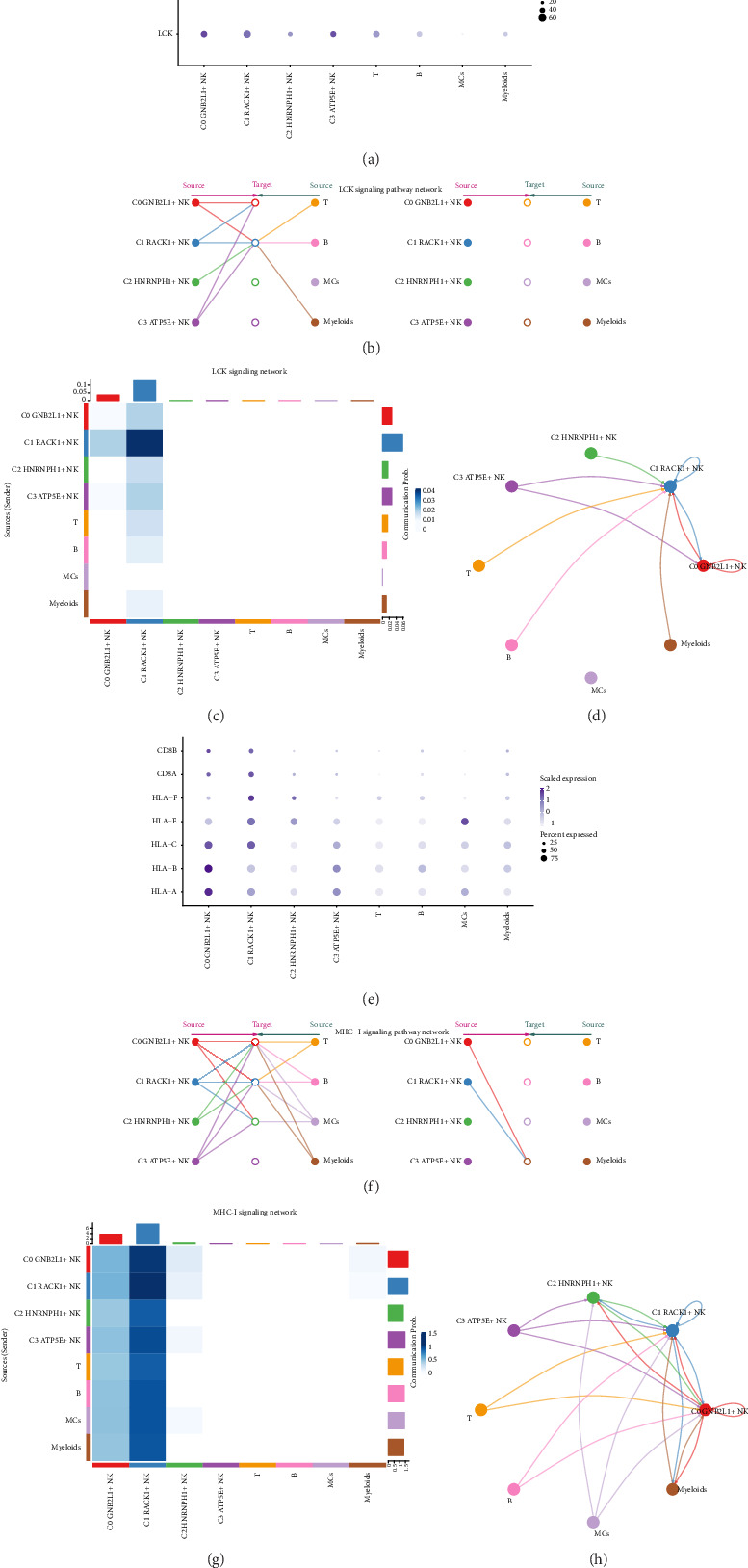
Analysis of cell–cell interactions in LCK and MHC-I signaling pathways. (a) The dot diagram showed the gene expression of four subgroups of NK cells and other cell types in LCK signaling pathway. The size of the dot indicated the percentage of gene expression in the cell types, and the depth of color indicated the level of gene expression. (b) The hierarchical diagram showed the interactions between NK cells and other cells in LCK signaling pathway. The solid circle and the hollow circle represented the source cell and the target cell types, respectively. The edge color of the middle circle was consistent with the signal sources. (c) Heatmap showed the centrality score of LCK signal path network and showed the relative importance of each cell group. (d) Cell interaction circle diagram with C1 RACK1+ NK cells as the source in LCK signaling pathway. (e) The dot diagram showed the gene expression of four subgroups of NK cells and other cell types in MHC-I signaling pathway. The size of the dot indicated the percentage of gene expression in the cell type, and the depth of color indicated the level of gene expression. (f) The hierarchical diagram showed the interaction between NK cells and other cells in MHC-I signaling pathway. The solid circle and the hollow circle represented the source cell and the target cell types, respectively. The edge color of the middle circle was consistent with the signal source. (g) Heatmap showed the centrality score of MHC-I signal path network and showed the relative importance of each cell group. (h) Cell interaction circle diagram with C1 RACK1+ NK cells as the source in MHC-I signaling pathway.

In order to explore the action pathway of LCK and MHC-I signal pathways, the two signal pathways were visualized and analyzed, respectively. The related genes on the LCK pathway with high expression of NK cell subgroup C1 *RACK1*+ NK cells was displayed by dot graph (Figure 6(a)).

When designating the eight recognized cell types in coronary plaque as the origin cells of LCK and the four cell types on the left side of Figure 6(b) as potential recipients, the hierarchical chart indicated that C1 *RACK1*+ NK cells were the only ones able to target LCK released by the majority of cell types (Figure 6(b)).

The algorithm determined the medium and influencer of intercellular communication through LCK signaling, known as “centrality measurement,” based on the relative importance of each cell type. The figure displayed that the NK cell subgroup C1 *RACK1*+ NK cells had the highest expression in the LCK signaling pathway (Figure 6(c)). A chord diagram (Figure 6(d)) illustrated the interaction between C1 RACK1+ NK cells and various intercellular ligands.

Similarly, the MHC-I signaling pathway was also visually analyzed to explore the expression of C1 *RACK1*+ NK cells in this signaling pathway. The related genes in MHC-I pathway of NK cell subgroup C1 *RACK1*+ NK cells were displayed by dot graph (Figure 6(e)). By designating all eight recognized cell types in coronary plaque as the origin cells of MHC-I, and identifying the four cell types on the left side of Figure 6(f) as possible target cells, the hierarchical chart indicated that solely C0 *GNB2L1*+ NK cells and C1 *RACK1*+ NK cells were able to target MHC-I released by all cell types (Figure 6(f)).

The algorithm determined the cell type's significance in intercellular communication through MHC-I signaling, labeling it as the “centrality measurement.” The figure indicated that the NK cell subgroup C1 *RACK1*+ NK cells exhibited the most significant expression on the MHC-I signaling pathway (Figure 6(g)). A chord diagram (Figure 6(h)) illustrated the interaction between C1 *RACK1*+ NK cells and various intercellular ligands. Using the C1 subgroup as a source revealed close relationships with various other cell types.

### 3.7. Analysis of Gene Regulatory Network of NK Cell Subgroups

**Figure 7:**
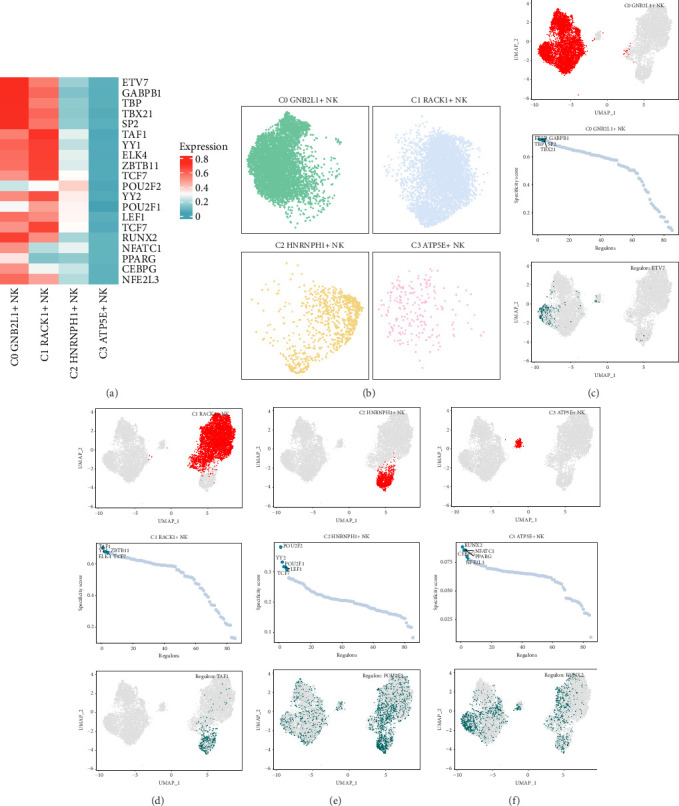
Analysis of gene regulatory network of NK cell subgroups. (a) Thermogram described the expression of Top5 TFs in four cell subgroups of NK cells. (b) The sectional view showed the distributions of four cell subgroups of NK cells after being treated by pySCENIC. (c–f) UMAP diagrams showed the distributions of NK cell subgroups (top). The ranking of regulators in four subgroups of NK cells based on regulon specificity score (RSS) (middle). And the distributions of the highest ranked regulator on the UMAP graphs (bottom).

SCENIC analysis was conducted to identify the key TFs present in the NK cell subgroup. PySCENIC has the capability to deduce the gene regulatory network for every subgroup of NK cells. According to the research results of cell type-specific regulatory activity, the most activated TFs in these myeloma cell subgroups included ETV7(C0 *GNB2L1*+ NK cells), TAF1(C1 *RACK1*+ NK cells), POU2F2(C2 *HNRNPH1*+ NK cells), and RUNX2(C3 *ATP5E*+ NK cells) (Figure 7(a)). The sectional diagrams also showed the expression of four subgroups of NK cells after pySCENIC (Figure 7(b)).

In order to display the gene expression more intuitively, scatter plots were also used. The UMAP plots (Figures 7(c), 7(d), 7(e), and 7(f)) ranked the regulators in the four cell subgroups of NK cells according to their regulon specificity scores (RSS). In UMAP plots, the four cell subgroups of NK cells were highlighted (red), and the activation scores of TFs with the highest activation in each subgroup were also displayed on UMAP plots (green).

### 3.8. Identification of TF Regulator Modules in NK Cell Subgroups

**Figure 8:**
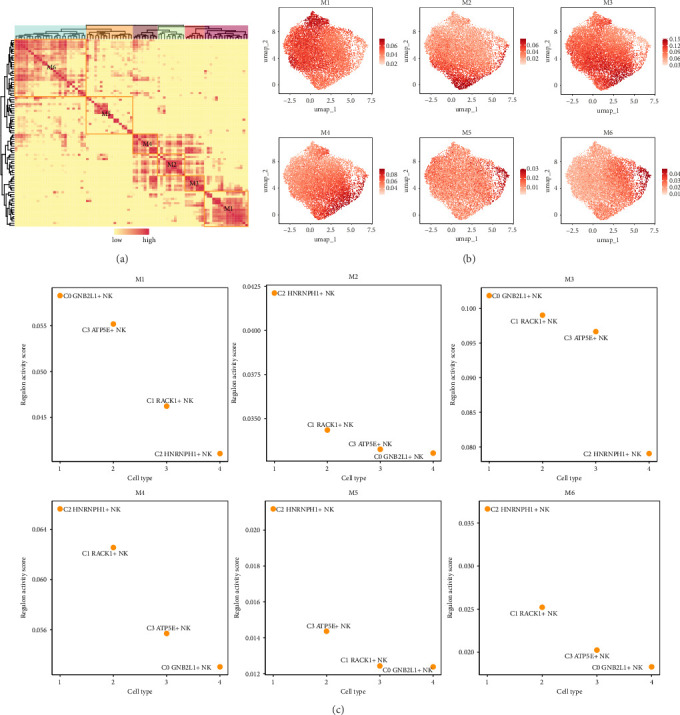
Identification of TF regulator modules in NK cell subgroups. (a) Six regulator modules (M1–M6) of NK cell subgroups were identified based on the matrix of connection specificity index (CSI). (b) UMAP diagrams showed the expression of 6 TF regulatory modules of NK cell subgroups. (c) Scatter plots demonstrated the transcriptional activity scores of four cell subgroups of NK cells on the M1–M6 modules.

In addition, we found six regulator modules of NK cell subgroups by using the matrix of connection specificity index (CSI). The group was split into six sections labeled as M1-M6 (Figure 8(a)). UMAP plots were used to show the expression of 6 TF regulatory modules of NK cell subgroups (Figure 8(b)). Scatter plots were utilized to display the transcription activity scores of four different cell subgroups of NK cells on modules M1-M6, as shown in Figure 8(c). The data indicated that the C2 *HNRNPH1*+ NK cell subgroup exhibited the highest expression level across the majority of modules, demonstrating a high regulation activity score. This was consistent with the results that the C2 *HNRNPH1*+ NK cell subgroup had the highest stemness.

### 3.9. Expression of NK Cell Subgroups on the Aging Pathway

**Figure 9:**
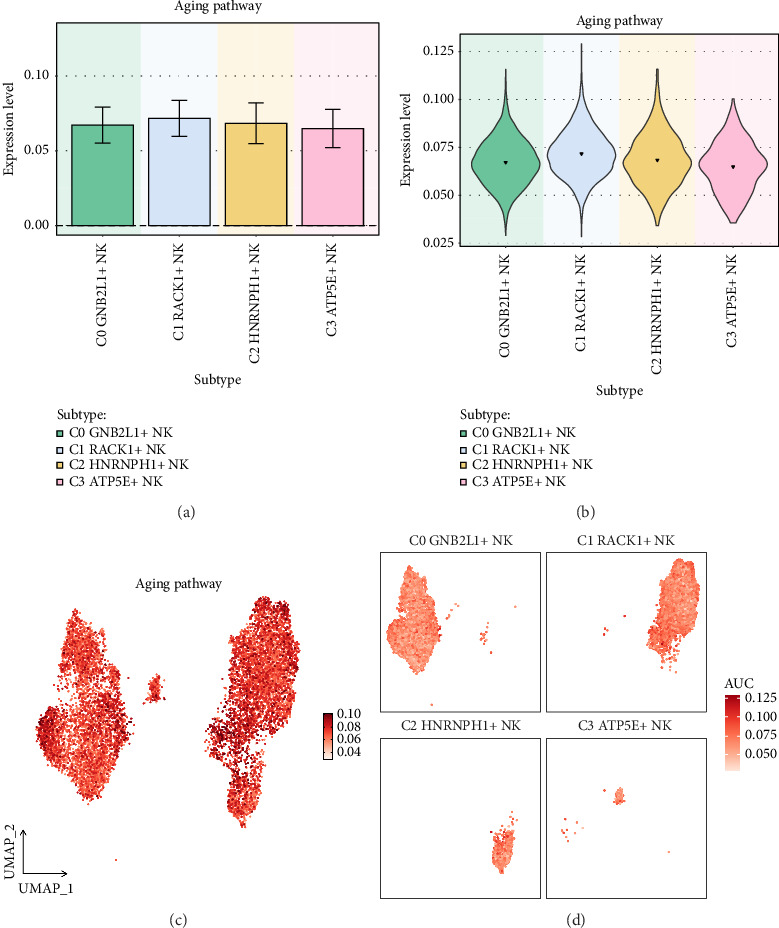
Expression of malignant NK cell subgroups on the aging pathway. (a-b) Histogram and violin plot showed the expression of malignant NK cell subgroups on the aging pathway. (c) UMAP plot showed the expression of malignant NK cell subgroups. (d) Facet plots showed the distribution of the four subgroups of malignant NK cells.

With age, blood vessels will gradually age and degenerate, creating conditions for the deposition of lipids and other substances. Aging causes a decline in the body's metabolic functions, such as abnormal lipid metabolism, which promotes plaque formation [60]. To investigate the potential correlation between coronary plaque formation and aging, the study showcased the expression of different subgroups of NK cells on the aging pathway. Four subgroups were visualized through histogram, box plot, UMAP plot, and facet plots, revealing that C1 *RACK1*+ NK cells had the highest expression on the aging pathway (Figures 9(a), 9(b), 9(c), and 9(d)).

## 4. Discussion

The diversity of cells and immune environment within coronary plaque was characterized using scRNA-seq technology. Coronary plaque exhibited the presence of NK cells, T-cells, B-cells, MCs, and myeloids. Inflammation has been shown in prior studies to play a crucial role in all stages of atherosclerotic plaque development, resulting in immune responses [61]. After clustering for dimensionality reduction, NK cells were identified as the most prevalent cell type. Previous research has demonstrated the significant involvement of NK cells in the development of coronary plaques [62], and the maintenance of plaque stability has been associated with NK cells [63].

Consequently, we opted for immune-associated NK cells and conducted dimensionality reduction clustering on NK cells and successfully distinguished four distinct cell subgroups. To explore the roles of these subgroups in coronary plaques, we extensively analyzed the characteristics of each cell subgroup from diverse viewpoints.

By utilizing slingshot, monocle, and CytoTRACE, we elucidated the differentiation process of NK cells along a pseudotime series trajectory. Our investigation specifically targeted the C1 *RACK1*+ NK cell subgroup. As to why this subgroup was selected, firstly, in predicted ordering by CytoTRACE, the expression level of C1 *RACK1*+ NK cells was the highest; that is, the differentiation ability was the strongest, which indicated that the cell stemness of this subgroup was the highest [64]. Secondly, in the pseudotime series analysis, a significant portion of the C1 *RACK1*+ NK cell subgroup was located at the initial position of the differentiation trajectory, further highlighting their strong differentiation potential. Given the close relationship between cell stemness and differentiation ability [65], the high cell stemness observed in this subgroup played a crucial role. Consequently, our subsequent studies were primarily focused on exploring the characteristics of the C1 *RACK1*+ NK cell subgroup.

Regarding the named genes of this subgroup, some previous studies have shown that *RACK1* interacted with some protein to form *RACK1* protein complexes and participated in the process of fibrosis after myocardial infarction [66, 67]. As a result, we deduced that the C1 *RACK1*+ NK cell subgroup was strongly associated with the advancement of coronary plaque. As for what role it played, we needed to analyze it from various aspects such as pseudotime series, cell communication, etc.

The slingshot pseudotime distribution revealed that the expression of C1 *RACK1*+ NK cell subgroup increased with the increase of pseudotime at first, and then decreased after reaching the peak value. This suggested that the presence of *RACK1*+ NK cells may contribute to the progression of coronary plaque formation (Kumric et al. 2020, Cited Pages). The pseudotime series analysis is helpful to understand the dynamic process of cell phenotype change during the formation of coronary plaque and provide direction for the study of the molecular mechanism of coronary plaque formation.

To explore the ligand–receptor relationship between C1 subgroup and other cells, we performed CellChat analysis. CellChat communication pattern analysis was utilized to uncover the coordinated response between C1 *RACK1*+ NK cell subgroup and other cells, aiming to investigate their interaction. The relationship between NK cell subgroups and other cell types was demonstrated in CellChat. Through the three patterns and their corresponding signal pathway expressions, LCK and MHC-I signal pathways could be obtained. During coronary plaque formation, LCK is recruited and activated after activated T-cells recognize antigens, initiating a series of downstream signal transduction events. This encourages T-cells to activate, proliferate, and differentiate into effector T-cells. MHC-I is upregulated by vascular wall cells and inflammatory cells. These cells process endogenous antigens into peptides that bind to MHC-I molecules and present them to CD8 + T-cells, which recognize and kill cells with abnormal expression of MHC-I antigen peptide complexes, triggering inflammatory responses and tissue damage [68].

To investigate the main TFs in different cell populations within coronary plaques, the SCENIC analysis was conducted, followed by gene regulatory network analysis for additional investigation. According to the CSI results, six main modules (M1-M6) were identified in the NK cell subgroups. In addition, we found that the TFs with the most activation among the NK cell subgroups in M1-M6 included ETV7(C0 *GNB2L1*+ NK cells), TAF1(C1 *RACK1*+ NK cells), POU2F2 (C2 *HNRNPH1*+ NK cells), and RUNX2(C3 *ATP5E*+ NK cells). In addition, the relationship between M1-M6 modules and cell subgroups was also demonstrated. The C2 *HNRNPH1*+ NK cell subgroup exhibited increased regulon activity scores across most modules, mirroring the finding that the C2 subgroup displayed the greatest stemness, as depicted in Figure 2(f) previously. We suspected that there may be some relationship between cell stemness and TFs that led to similar results. In previous studies, it has been proposed that TFs were essential for cell stemness by enhancing gene expression in transcription condensates formed by mediators such as coactivators and RNA [69]. Therefore, we concluded that TFs have a positive correlation with cell stemness.

Finally, the relationship between the C1 *RACK1*+ NK cell subgroup and the aging pathway was discussed. The relationship between the two was studied because previous studies showed that the probability of coronary plaque increases with age [70], and the results showed that C1 *RACK1*+ NK cell subgroup was most expressed on the aging pathway. As mentioned above, the expression of this subgroup increased with the development of coronary plaque, suggesting that there may also be a promoting relationship between aging pathway and coronary plaque. This was of great significance for us to carry out the prevention of coronary plaques. The interaction of key subgroups with the immune microenvironment affects the stability and progression of plaques. The single-cell approach provides a powerful tool for studying the relationship between cell subsets and their immune microenvironment in coronary plaque formation, which helps to further understand the plaque formation mechanism and provide a target for developing new therapeutic strategies.

In a word, we analyzed NK cells which had a high proportion in coronary plaques in multiple aspects through scRNA seq, and found an important subgroup of them, the C1 *RACK1*+ NK cell subgroup, and analyzed them from multiple levels such as cell stemness, pseudotime trajectory, cell-to-cell communication, TFs, and the relationship with aging pathway to explore further understanding of coronary plaques. However, they are all in vitro experiments and have certain limitations, such as the difficulty of completely simulating the complex physiological environment in vivo in the in vitro environment, possible omissions in the detection of intercellular communication, and failure to fully consider the complex regulatory mechanisms of TFs in vivo. In the future, we will validate the key genes and molecular mechanisms obtained in vitro in animal models and human tissue samples to make our results more convincing. The study offered fresh perspectives on how to treat individuals with coronary artery blockages. This is crucial in inhibiting the development of coronary plaque. This article has two limitations, the first limitation of this study is that the number of samples selected was small, and it was only for 12 coronary plaque patients. In a small sample, individuals vary widely in lifestyle habits (such as diet, exercise, smoking, etc.) and environmental exposures (such as pollution, chemical exposure) that can interfere with the process of coronary plaque formation. Small samples are more susceptible to random factors, resulting in less reproducibility and robustness of research results. In the future, we will use larger data sets and integrate relevant single-cell studies for meta-analysis, to make our findings more reliable. The second limitation is that our results have not been validated experimentally, and we will further verify the experimental results in future work. We have planned to conduct flow cytometry, qPCR, and immunohistochemistry experiments to validate the role of C1 *RACK1*+ NK cells in the future. In the follow-up study, we will further verify the role of *RACK1*+ NK cells in coronary plaque, hoping that this study will contribute to the current understanding of clinical treatment of coronary plaque [71], and have an impact on treatment decision-making and long-term monitoring.

The results of our study have potential implications for coronary plaque treatment. The identification of C1 *RACK*1+ NK cells and their associated factors provides new targets for therapeutic strategies. For example, we could potentially develop drugs or immunotherapies that target the specific functions or interactions of these cells. Understanding the microenvironment also allows for the design of more personalized treatment regimens. For instance, patients with a particular abundance or activity of C1 *RACK*1+ NK cells could be treated with therapies tailored to modulate their immune response. Additionally, strategies to promote plaque stability or regression could be developed based on our insights into the cellular and molecular mechanisms.

## 5. Conclusion

Although these data represent an important progress in the cognition of coronary plaque, the limitations of current work should be taken into account in future research. Initially, the size of the sample is quite limited, and conducting research on a larger scale will be beneficial in validating and broadening our findings. Secondly, we need to further verify our findings on coronary plaque samples, including the accuracy of the important subgroup C1 *RACK1*+ NK cells we selected.

In a word, our research provides the cellular microenvironment and related important subgroups of coronary plaque in the process of plaque occurrence and progress. Studying NK cells and different cell clusters related to the beginning and advancement of coronary plaque offers possible treatment options and valuable insights into the occurrence and progression of coronary plaque.

## Data Availability

The data for this study come from the Gene Expression Omnibus (GEO) (https://www.ncbi.nlm.nih.gov/geo/) database. The GEO accession is GSE196943. All the data in this paper support the results of this study. The raw data and code have been uploaded to https://www.ncbi.nlm.nih.gov/geo/query/acc.cgi?acc=GSE196943.

## Supporting Information

Supporting Information 1 Supporting Figure 1: InferCNV explored single-cell RNA-seq data from NK cells to distinguish NK cells.

Supporting Information 2 Supporting Figure 2: UMAP, violin, and pseudotime scatter plots were used to show the distribution of named genes of four cell subgroups on the pseudotime series.
