# Supplementary figures and images for: To Explore the Key Subgroup and Their Immune Microenvironment During the Formation of Coronary Plaque With scRNA-seq
Source: Cardiol Res Pract. 2025 May 24;2025:3221767. doi: 10.1155/crp/3221767 (PMC12126265; doi:10.1155/crp/3221767)

# inferCNV

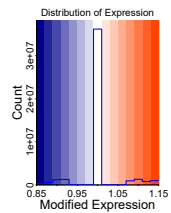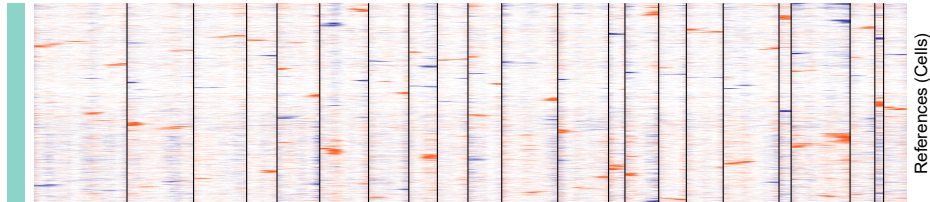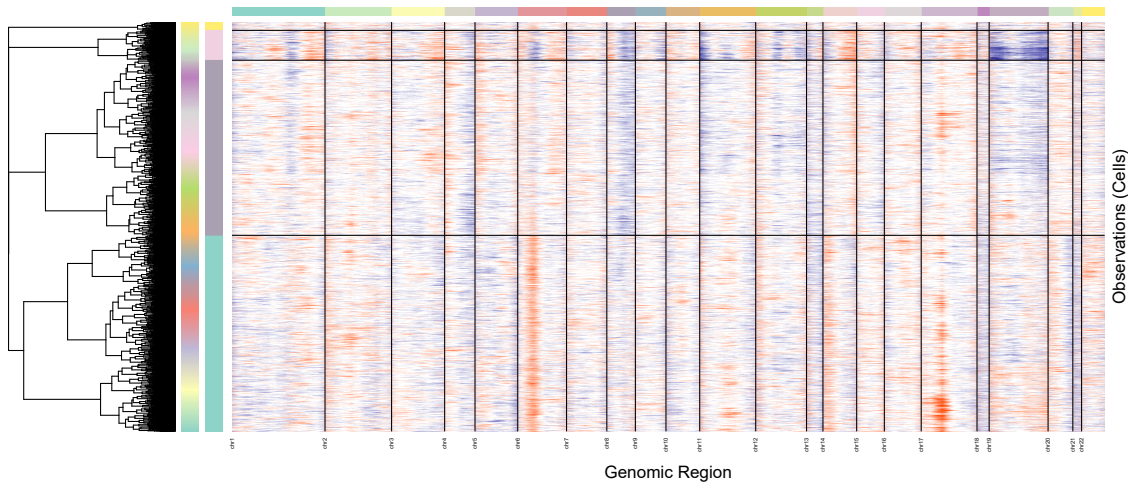

T

■ C0 GNB2L1+ NK 
 ■ C1 RACK1+ NK 
 ■ C2 HNRNPH1+ NK 
 ■ C3 ATP5E+ NK

Supplement: Supporting Information 1 — Supporting Figure 1: InferCNV explored single-cell RNA-seq data from NK cells to distinguish NK cells. [file 3221767.f1.pdf]

A

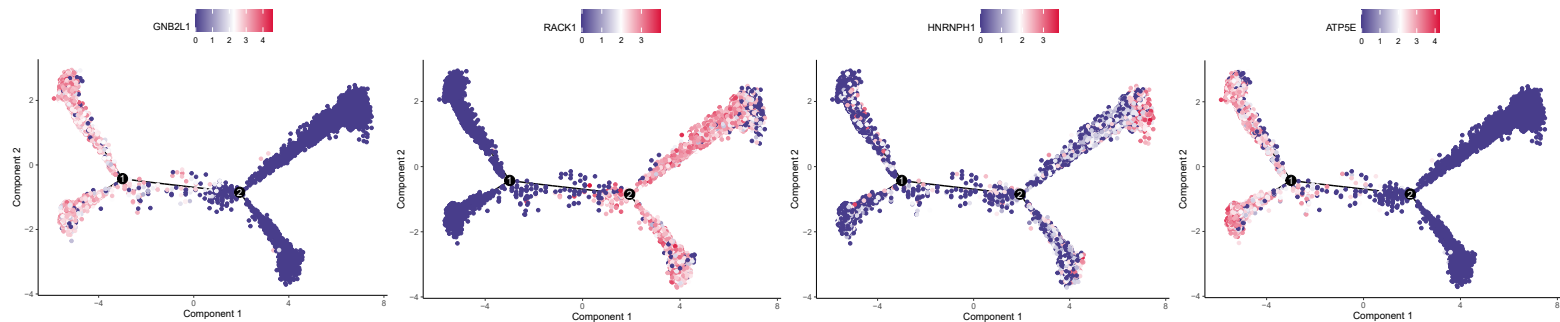

B

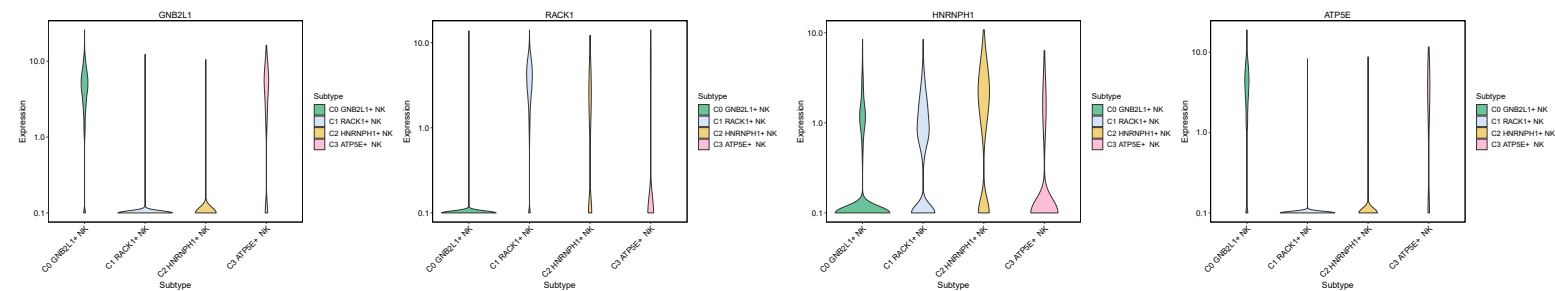

C

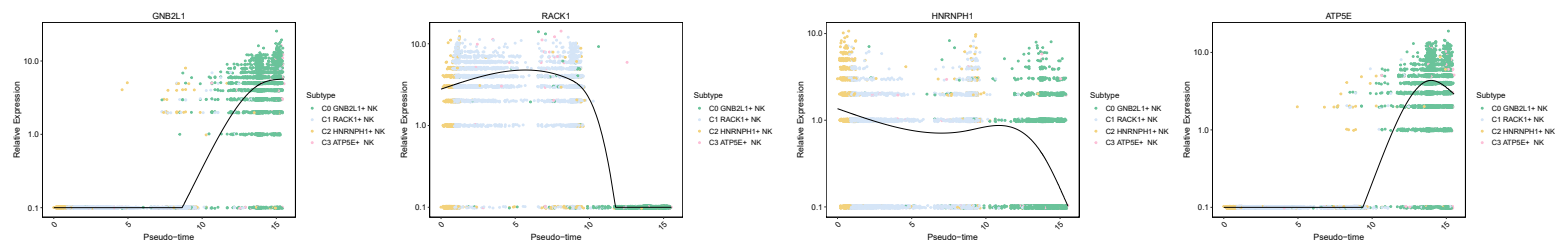

Supplement: Supporting Information 2 — Supporting Figure 2: UMAP, violin, and pseudotime scatter plots were used to show the distribution of named genes of four cell subgroups on the pseudotime series. [file 3221767.f2.pdf]
